# Bradykinin Type-2 Receptor Expression Correlates with Age and Is Subjected to Transcriptional Regulation

**DOI:** 10.1155/2012/159646

**Published:** 2011-10-03

**Authors:** Inka Liesmaa, Naotaka Shiota, Jorma O. Kokkonen, Petri T. Kovanen, Ken A. Lindstedt

**Affiliations:** ^1^Wihuri Research Institute, Kalliolinnantie 4, 00140 Helsinki, Finland; ^2^Department of Cardiology, Jyväskylä Central Hospital, 40620 Jyväskylä, Finland

## Abstract

Accumulating work in experimental animals suggests that bradykinin (BK) exerts cardioprotective effects via bradykinin type-2 receptors (BK-2Rs). In human end-stage heart failure, BK-2Rs are significantly downregulated by mechanisms that have remained elusive. Heart tissues from idiopathic dilated cardiomyopathy (IDC; *n* = 7), coronary heart disease (CHD; *n* = 6), and normal patients (*n* = 6) were analyzed by RT-PCR, SSCP, and Western blotting. In normal and IDC hearts, BK-2R expression increased with age, with a lower relative increase in IDC hearts. BK-2R mRNA and protein levels showed a positive linear correlation, suggesting transcriptional regulation. Two known BK-2R promoter polymorphisms, −58T/C and −9/+9, were found to be present in the study population. The allelic frequencies for the C-allele in −58T/C were 0.58 in normal and CHD hearts and 0.81 in IDC hearts. Furthermore, the allelic frequencies for the −9 and +9 alleles were 0.42 and 0.58 in normal hearts and 0.64 and 0.36 in IDC hearts, respectively. All analyzed CHD hearts were homozygous for the −9 allele. Thus, the expression of cardioprotective BK-2Rs in human hearts is increased with age in normal and IDC hearts and may be regulated on the transcriptional level. Moreover, comparison of normal subjects and patients with failing hearts revealed different allelic frequencies in each of two known BK-2R gene polymorphisms.

## 1. Introduction

Accumulating data indicate that bradykinin (BK) exerts cardioprotective effects, which include both protection of the myocardium from ischemia-reperfusion injuries [1–4] and prevention of left ventricular hypertrophy (LVH) and heart failure [5–7]. The cardioprotective effects are mainly mediated by the bradykinin type-2 receptor (BK-2R), a member of the G protein-coupled receptor superfamily, resulting in vasodilatation, that is release of nitric oxide by endothelial cells [4, 5, 8], together with both antiproliferative [6, 9] and antihypertrophic [7, 10] effects on fibroblasts and myocytes. 

In human end-stage heart failure, due to either idiopathic dilated cardiomyopathy (IDC) or coronary heart disease (CHD), the amount of cardioprotective BK-2Rs is significantly decreased [11]. The observed reduction in BK-2R expression associated with a decrease in endothelial nitric oxide synthase (eNOS) in the failing hearts and with an increase in the level of fibrosis in IDC hearts [11]. 

Although the molecular mechanisms regulating the expression of BK-2Rs are largely unknown, it is generally thought that the main regulatory mechanism lies at the gene expression level [12]. An altered frequency of a promoter (−58T/C) polymorphism in the BK-2R gene has been described in hypertensive African-Americans [13], Japanese [14], and Chinese [15] population, suggesting that a decreased transcriptional activity in the BK-2R promoter may be involved in essential hypertension. Accordingly, a partial genetic deficiency (heterozygous) of BK-2Rs in mice is already sufficient for the onset of mild LVH [16]. Recent data by Duka et al. have indicated that both BK-1Rs and BK-2Rs contribute to the maintenance of normal blood pressure in Wistar rats, but that one receptor can compensate for inhibition of the other, and that a chronic inhibition of both receptors resulted in significant upregulation of related vasoactive systems [17]. Thus, a decreased expression of BK-2Rs may be compensated with an increased expression of BK-1Rs. Furthermore, the +9 allele of a −9/+9 exon 1 polymorphism of the BK-2R is strongly associated with an increased LV growth response among normotensive males undergoing physical training [18], and with impaired LV mass regression during antihypertensive treatment [19]. In contrast, Fischer et al. [20] found no relation between the −9/+9 exon 1 polymorphism and the prevalence of myocardial infarction (MI) nor LV function after MI. However, it is possible that patients with end-stage heart failure have a genetically determined fault in their BK-2R expression, which could affect the balance between cardiotoxic and cardioprotective systems, as well as their individual responses to medical treatments. 

In the present study, we have analyzed the age-related regulation of BK-2R expression and the occurrence of two known BK-2R promoter polymorphisms in normal and failing human hearts.

## 2. Materials and Methods

### 2.1. Preparation of Human Heart Samples

Normal heart samples (*n* = 6) were obtained from left ventricles of organ donors who had no history of cardiac disease and had been excluded from organ donation because of age, body size, or tissue type mismatch. The cause of death in the control patients was subarachnoid hemorrhage (SAH). Sample collection was performed in accordance with the Helsinki Declaration, local legislation, and an institutional review board approved the study protocol. The informed consent was given by the next of kin prior to organ donation. Failing left ventricles were harvested at the time of cardiac transplantation from 13 patients with end-stage heart failure (New York Heart Association functional class IV) due to either IDC (*n* = 7) or CHD (*n* = 6) at the University Central Hospital, Helsinki, Finland. All patients were treated with a combination of drugs including *β*-blockers, ACE inhibitors, loop-diuretics, digoxin, and spironolactone. After excision, the heart tissues were immediately frozen in liquid nitrogen and stored at −70°C. Left ventricle myocardium devoid of visible scar tissue was used in the experiments. The clinical characteristics of the patients in this study are shown in Table 1. An institutional review board approved the use of failing human heart samples.

### 2.2. Competitive RT-PCR

Total RNA was isolated from human heart samples using an ultra-pure TRIzol reagent (GIBCO BRL), and an RNeasy Mini Kit (QIAGEN). One microgram of purified total RNA was transcribed into cDNA, using a Superscript TM pre-amplification system (GIBCO BRL). The primers were as follows: BK-2R: 5′-CACCATCTCCAACAACTTCG (S), 5′-GGTAGCTGATGACACAAGCG (AS); GAPDH: 5′-ACCACAGTCCATGCCATCAC (S), 5′-TCCACCACCCTGTTGCTGTA (AS). The competitor DNA for the BK-2R was obtained by inserting a 129 bp external DNA fragment into the SacI site. The use of equal amounts of mRNA in the RT-PCR assays was confirmed by analyzing the expression levels of glyceraldehyde-3-phosphate dehydrogenase (GAPDH) (data not shown). The PCR product was verified, by DNA sequencing, to represent the corresponding target and quantitated with a Gel Doc 2000 gel documentation system (Bio-Rad). The logarithm of the target-to-competitor ratio was plotted against the logarithm of the competitor DNA-molecules [11].

### 2.3. Detection of a −/+9 Polymorphism in Exon 1 of BK-2R Gene by PCR

Genomic DNA was isolated from human heart tissue using an RNA/DNA Mini Kit (Qiagen) and stored in TE buffer (pH 8.5) in aliquots at −20°C.

Isolated genomic DNA was subjected to PCR using primers spanning the insertion/deletion (−/+9 bp) site in exon 1 of the BK-2R gene. The primers were as follows: BK-2R: 5′-CAAAGATGAGCTGTTCCCGCC (S), 5′-GGGAACTTTTCCCAACTCCCC (AS). The PCR was run at *T*_*m*_ = 66°C for 40 + 1 cycles and the obtained PCR products were separated on a 3% MetaPhor agarose gel (BMA).

### 2.4. Detection of a −58T/C BK-2R Promoter Polymorphism by PCR and SSCP

Genomic DNA was isolated as described above and subjected to PCR using following primers: BK-2R: 5′-AGGAGTGCAGAGCTCAGCTGGAG (S), 5′-TCGGAGCCCAGAAGCCAGAG (AS). The obtained PCR products were denatured by heating at 94°C for 3 minutes in 95% formamide, 10 mM NaOH, 0.25% bromphenol blue, and 0.25% xylene cyanol, and then were rapidly cooled on ice. The denatured PCR samples were mixed with 9 parts of SSCP Sucrose Dye (10 g sucrose, 62.5 mg BPB.XC, 0.5 ml 0.5 M EDTA (pH 8.0) in 25 ml dH_2_O), and run on a 14% acrylamide/bis (29 : 1) gel (BIORAD) containing 10% (v/v) 2x concentrated MDE gel solution (BMA) for 5 min at 500 V, and then for 19 hours at 250 V at room temperature. The gel was stained with SYBR Gold nucleic acid gel stain (Molecular Probes) for 30–40 min and analyzed with a Gel Doc 2000 gel documentation system (Bio-Rad).

## 3. Statistics

The data was subjected to linear regression analysis. Statistical significance was accepted at *P* < 0.05.

## 4. Results

### 4.1. BK-2R Expression Increases with Age in Normal and IDC Hearts

**Figure 1:**
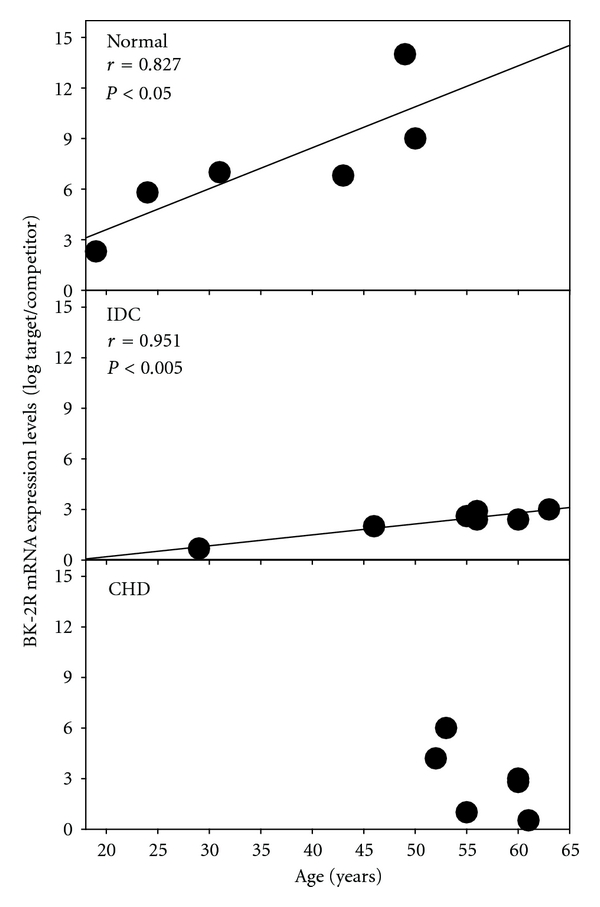
BK-2R mRNA expression correlates with age in normal and failing (IDC) hearts. The level of BK-2R mRNA expression was plotted against age of normal, IDC, and CHD hearts, and the correlation was calculated. *P* < 0.05 was considered significant.

By simple linear regression analysis, a strong positive correlation (*r* = 0.827; *P* < 0.05) was found between the level of BK-2R mRNA expression and age in normal human hearts (Figure 1, upper panel), suggesting that normal hearts adapt to age-related changes by increasing their expression of cardiac BK-2Rs. In addition, a significant positive correlation (*r* = 0.951; *P* < 0.001) was also seen between the level of BK-2R mRNA expression and age in the IDC hearts (Figure 1, middle panel). However, the relative increase in BK-2R mRNA expression, that is the slope of the regression curve, was significantly lower in the IDC hearts than in the normal hearts. Surprisingly, a mild negative correlation (*r* = 0.509) was found between BK-2Rs mRNA and age in the CHD hearts (Figure 1, bottom panel), suggesting a disease-dependent suppression of BK-2R expression. On account of the nature of the disease, all the CHD hearts were from patients older (>50 years) than those in the normal group.

### 4.2. Linear Correlation between BK-2R mRNA and Protein Expression

**Figure 2:**
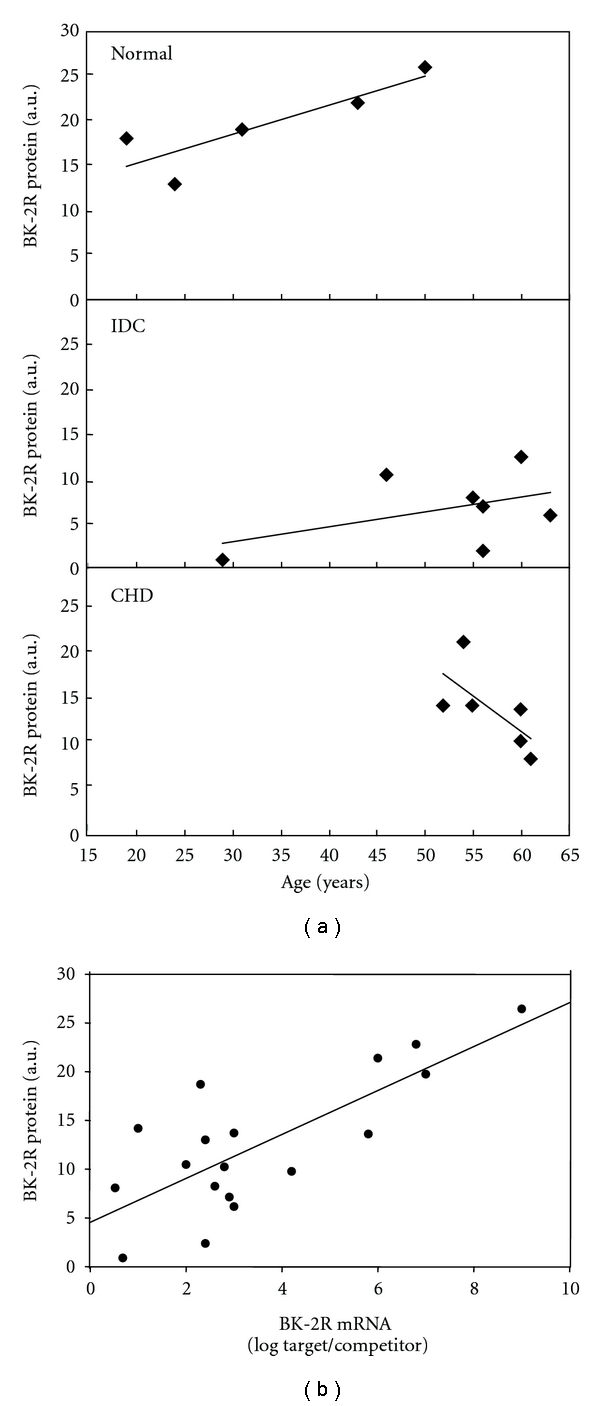
(a) BK-2R protein levels in normal and failing hearts show a linear trend (not significant) when plotted against age. (b) BK-2R mRNA expression levels plotted against BK-2R protein levels show a positive linear correlation (*R* = 0.761, *P* < 0.001), suggesting that BK-2R expression may be regulated on the transcriptional level.

By further plotting the BK-2R protein levels against age of the patients (Figure 2(a)) and the BK-2R mRNA levels against the receptor protein levels of both normal and failing hearts (*n* = 18) (Figure 2(b)), we found a positive linear correlation (*R* = 0.761, *P* < 0.001) between the mRNA and protein levels. This result suggests that the expression of BK-2Rs in both normal and failing human hearts may be regulated on the transcriptional level, rather than on the translational level. 

### 4.3. BK-2R Polymorphism in Failing Human Hearts

To further analyze the possible transcriptional mechanisms involved in the observed downregulation of BK-2R expression in failing hearts, we determined the presence of two polymorphisms, a −58T/C promoter polymorphism and a 9 base pair (bp) exon 1 deletion/insertion polymorphism, both previously known to affect the BK-2R expression levels (Table 1). The characteristics of the analyzed patients as well as the presence of the two analyzed BK-2R polymorphisms, are described in Table 1. As shown in Tables 1 and 2, the genotypic frequency of CC in the IDC hearts (71%) was significantly increased, as compared to the normal hearts (33%). In contrast, the frequency of the TT genotype was found to be similar in both IDC (14%) and normal (17%) hearts, whereas the TC genotype dominated in the normal hearts (50%), as compared to the IDC hearts (15%). The genotypic frequencies in the CHD hearts were identical to the normal hearts, that is, 33% were CC, 17% were TT, and 50% were TC. Furthermore, the allelic frequencies of the −58T/C promoter polymorphism were 0.58 for the C-allele and 0.42 for the T-allele in both normal and CHD hearts. In contrast, the allelic frequencies in IDC hearts were 0.81 for the C-allele and 0.19 for the T-allele. In terms of the 9-bp exon 1 deletion/insertion polymorphism, we found that all patients in the CHD group (100%) were homozygous for the deletion (−9/−9) (Table 2). In contrast, in the normal group, only 17%, and in the IDC group, 29% were homozygous for the 9 bp deletion. Among the IDC hearts, 71% were heterozygous, whereas, in the normal hearts, 50% were found to be heterozygous (−9/+9) and 33% were homozygous for the +9 allele.

## 5. Discussion

Here we show that a linear relationship between BK-2R mRNA and protein expression exists in normal and failing human left ventricles, suggesting that the expression of cardioprotective BK-2Rs in human myocardium may be regulated on the transcriptional level, rather than on the translational level. In addition, in normal and IDC hearts, the BK-2R expression was found to correlate positively with age, but the relative increase was clearly lower in the IDC hearts than in the normal hearts. The increased presence of the C-allele in the IDC hearts may explain the age-related lower relative increase in BK-2R expression in IDC hearts, as compared to normal hearts. These results suggest that the human heart adapts to age-related changes in heart function [21] by upregulating the expression of cardioprotective BK-2Rs. This is consistent with previous studies performed with BK-2R KO mice, which in the absence of BK-2Rs show an accelerated ageing phenotype [16, 22]. Moreover, in a recent study, BK was shown to protect against ROS-mediated DNA damage and reduce endothelial cell senescence via BK-2R and NO-dependent pathway, supporting an important role for BK and BK-2Rs in ageing processes [23].

In a recent study, a correlation between age and the expression of BK-1Rs and BK-2Rs has also been observed in male Brown Norway rats [24]. The authors showed that the expression of BK-2Rs was decreased in rat hearts as a function of age, whereas the expression of BK-1Rs was increased. Hence, they suggested that the age-related decrease in BK-2Rs is cardiotoxic and that the increased expression of BK-1Rs may be a compensatory effect. Similarly, in our previous studies, we observed an age-related decrease in both mRNA and protein expression of BK-2Rs in normal Wistar-Kyoto rats (WKYs) [25]. However, in contrast to normotensive WKYs, spontaneous hypertensive rats (SHRs), that is, rats with a hypertensive phenotype, showed an age-related increase in BK-2R mRNA expression. Interestingly, also BK-2R protein levels increased in the SHRs during the first half of their lifespan, that is, during the LVH stage, but were significantly decreased in the failing rat hearts [25]. In our present human studies, the expression of BK-2Rs increased with increasing age in both the control and the IDC group, whereas a relative decrease in BK-2Rs was observed in the CHD study group. 

It seems evident that LVH may develop as a compensatory mechanism during the early stages of the disease, whereas the later stages are characterized by a maladaptive loss of myocytes with subsequent fibrosis, LV dysfunction, and heart failure. Thus, if BK-2Rs are an essential part of a local cardioprotective system, a logical adaptive response to an ongoing pathological process would be an increased expression of BK-2Rs. Therefore, it is possible that BK-2R expression is upregulated during LVH, and that the observed receptor downregulation occurs only later, at the stage of heart failure. Similarly, the increased expression of BK-2Rs with increasing age in normal human hearts may occur as an adaptive response to an age-related physiologic process of disease-independent fibrosis [23]. It therefore seems unlikely that the observed down-regulation of BK-2Rs in the failing hearts is due merely to the greater age of the patients. This, however, is clearly different from normotensive rats, in which the expression of BK-2Rs is decreased with increasing age [24, 25]. The increased levels of fibrosis observed in the IDC hearts [11] in relation to a reduction in the number of myocytes may partly also explain the reduced amount of BK-2Rs in the IDC hearts, however; this may not hold true for the CHD hearts, since no increase in fibrosis was observed in CHD hearts [11].

Recent studies have suggested that patients developing LVH and heart failure may have a genetic background in which their expression levels of cardioprotective BK-2Rs are significantly weaker than in normal hearts, or completely lacking. Indeed, the C allele of the −58T/C promoter polymorphism in the BK-2R gene has been associated with the occurrence of essential hypertension in several ethnic groups, including African Americans [13], Japanese [14], and Chinese [15] populations. Our present results also suggest that the expression of BK-2Rs in IDC hearts is associated with the C allele of the –58T/C promoter polymorphism and are the first to suggest that this allele is accumulated in IDC patients and may partially explain the observed reduction in BK-2R expression in this patient group [11]. In contrast, the distribution of the C allele in CHD hearts is similar to the distribution in normal hearts, although the expression of BK-2Rs appears negatively correlated with age in the CHD group.

Military trainees, which were homozygous for the −9 allele and underwent physical training, did not develop exercise-induced myocardial hypertrophy to a similar extent as heterozygous subjects or homozygous for the +9 allele [18]. This result suggests that the −9 allele suppresses physiological LV hypertrophy induced by physical training, whereas the +9 allele may associate with increased physiological hypertrophy, and possibly also with pathological hypertrophy. Indeed, it was recently shown that patients with a +9/+9 genotype and suffering from essential hypertension responded poorly in LV mass regression, independent of blood pressure reduction or treatment, as compared to other genotypes [19]. In the present work, none of the IDC and CHD hearts were homozygous for the +9/+9 genotype, whereas 33% of the normal hearts were +9/+9 genotypes. This result contradicts previous studies, in which the +9/+9 genotype has been linked to increased exercise-induced physiological LV hypertrophy in normotensive subjects and to poor LV mass regression during antihypertensive treatment. However, the question remains whether the mechanisms involved in the progression of physiological and pathophysiological LV hypertrophy diverge, and whether also the cardioprotective mechanisms vary depending on the initial cause of the disease? Similarly, the contribution of BK-2R polymorphisms in the development of LV hypertrophy and heart failure may differ between CHD hearts and IDC hearts. These statements remain to be considered in future experiments.

## 6. Limitations of the Study

The number of human heart samples in this study is too low to allow any conclusions regarding the general distribution of the studied polymorphisms in failing hearts on the population level. Future studies should aim at analyzing the correlation between IDC and CHD patients and specific promoter polymorphisms capable of affecting the expression of BK-2Rs in a large clinical setting. However, due to the advancement of various preventive treatment modes of coronary heart disease, particularly the use of statins, the number of CHD patients subjected to transplantations per year has dramatically dropped. Thus, most of the patients undergoing heart transplantation are diagnosed with primary myocardial disease. Also, acute coronary syndromes hardly lead to massive myocardial necrosis any more. Finally, patients with multiple myocardial infarctions and ensuing end-stage heart failure are also very rare, and if patients have a severe heart failure due to CHD, they are too old for heart transplantation. 

## Figures and Tables

**Table 1:** BK-2R polymorphism in human hearts. The presence of two BK-2R gene polymorphisms, −58T/C and −9/+9, was analyzed in normal and failing human hearts according to the protocol described in Section 2.

Subject no.	Etiology	Sex	Age	Polymorphism	
				−58 T/C	+9/−9
1	Normal	M	19	T/C	+9/−9
2		M	43	T/C	+9/+9
3		M	49	C/C	+9/−9
4		M	31	T/T	−9/−9
5		M	40	T/C	+9/+9
6		F	50	C/C	+9/−9

7	CHD	M	61	T/C	−9/−9
8		M	60	T/T	−9/−9
9		M	55	C/C	−9/−9
10		M	52	T/C	−9/−9
11		M	60	C/C	−9/−9
12		M	54	T/C	−9/−9

13	IDC	M	29	C/C	−9/−9
14		M	60	C/C	−9/−9
15		M	46	C/C	+9/−9
16		M	56	C/C	+9/−9
17		M	55	T/T	+9/−9
18		M	56	T/C	+9/−9
19		M	63	C/C	+9/−9

**Table:** (a)

Polymorphism	Genotype	Normal	IDC	CHD
−58T/C	T/T	17.0%	14.0%	17.0%
	T/C	50.0%	15.0%	50.0%
	C/C	33.0%	71.0%	33.0%

−9/+9	−9/−9	17.0%	29.0%	100%
	−9/+9	50.0%	71.0%	0%
	+9/+9	33.0%	0%	0%

**Table:** (b)

Polymorphism	Allele	Frequency			
		Normal	IDC	CHD	Average

−58T/C	C-allele	0.58	0.81	0.58	0.66
	T-allele	0.42	0.19	0.42	0.34

−9/+9	−9-allele	0.42	0.64	1.00	0.69
	+9-allele	0.58	0.36	0.00	0.31
